# Supplementary material for: First genomic insights into members of a candidate bacterial phylum responsible for wastewater bulking
Source: PeerJ. 2015 Jan 27;3:e740. doi: 10.7717/peerj.740 (PMC4312070; doi:10.7717/peerj.740)
Supplement: Supplemental Information 1 [file peerj-03-740-s001.docx]

**Supplementary Notes**

**Description of new taxa.**

**Description of ‘*Candidatus* Moduliflexus flocculans’**

‘*Candidatus* Moduliflexus flocculans’ [L. verb. *modulor*, to attune; L. masc. adj. *flexus*, bent, winding; N.L. masc. n. *Moduliflexus*, to indicate long flexible rods that are attuned to their surroundings. N.L. part. adj. *flocculans*, flocculating: long flexible rods that are attuned to their surroundings often causing flocculate (bulking) sludge]. Not cultivated. Belongs to the uncultured bacterial phylum *Modulibacteria* phylum nov. Represented by the population genome UASB14 (accession DF820455-DF820462) obtained from metagenomes of methanogenic granular sludge from an industrial UASB reactor system. They are Gram-negative, filamentous, strictly anaerobic fermenters capable of gliding motility. They have a large genome (7.1 Mb) containing an extensive number of receiver and response regulatory genes, amongst the highest number (and proportion) identified in any bacterial genome to date. They may generate energy through the glycolytic Embden-Meyerhof (EMF) pathway and the fermentation of amino acids and sugars. Habitat is a UASB granular sludge.

**Description of *Moduliflexaceae*, fam. nov.**

*Moduliflexaceae* [N.L. masc. n. *Moduliflexus*, type candidatus genus of the family; -*aceae* ending to donate a family; N.L. fem. pl. n. *Moduliflexaceae* family of the candidatus genus *Moduliflexus*]. They are Gram-negative, filamentous, strictly anaerobic fermenters that are capable of gliding motility. Type genus: *Candidatus* Moduliflexus.

**Description of *Moduliflexales* order nov.**

*Moduliflexales* (N.L. neut. n. *Moduliflexus* type genus of the order; -*ales* ending to donate an order; N.L. fem. pl. n. *Moduliflexales* the order of the genus *Moduliflexum*). The description is the same as for the family *Moduliflexaceae*. Type family: *Moduliflexaceae* fam. nov.

**Description of *Moduliflexia* classis nov.**

*Modulilexia* (N.L. neut. pl. n. *Moduliflexia*). The description is the same as for the family *Moduliflexaceae*. Type order: *Moduliflexales* order. nov.

**Description of ‘*Candidatus* Vecturithrix granuli’**

‘*Candidatus* Vecturithrix granuli’ [N.L. fem. n. *vectura*, transportation; -i- connecting vowel; Gr. fem. n. *thrix*, hair; N.L. fem. n. *Vecturithrix*, to indicate long rods that have a large number of transporter related systems in their genome. L. neut. gen. n. *granuli* of granule: long rods that has a number of transporter related systems in their genome, living in granular sludge] Not cultivated. Belongs to the uncultured bacterial phylum *Modulibacteria* phylum nov. Represented by the population genome UASB270 (accession DF820463-DF820483), which was obtained from metagenomes of methanogenic granular sludge from a UASB reactor system. They are Gram-negative, filamentous, strictly anaerobic fermenters that may be capable of gliding motility. They have a large genome (8.4 Mb) containing an extensive number of receiver and response regulatory genes, and transporter related gene systems. They may produce acetate, ethanol, lactate, and hydrogen (and possibly propionate) as fermentative end products, likely generating energy through the glycolytic Embden-Meyerhof (EMF) pathway and the fermentation of amino acids and sugars. Habitat is a UASB granular sludge.

**Description of *Vecturitrichaceae* fam. nov.**

*Vecturatrichaceae* [N.L. fem. n. *Vecturithrix*, type candidatus genus of the family; -*aceae* ending to donate a family; N.L. fem. pl. n. *Vecturitrichaceae* family of the candidatus genus *Vecturithrix*]. They are Gram-negative, filamentous, strictly anaerobic fermenters that may be capable of gliding motility. Type genus: *Candidatus* Vecturithrix.

**Description of *Vecturitrichales* order nov.**

*Vecturatrichales* (N.L. fem. n. *Vecturithrix*, type genus of the order; -*ales* ending to donate an order; N.L. fem. pl. n. *Vecturatrichales* the order of the genus *Vecturithrix*). The description is the same as for the family *Vecturitrichaceae*. Type family: *Vecturitrichaceae* fam. nov.

**Description of *Vecturitrichia* classis nov.**

*Vecturitrichia* (N.L. neut. pl. n. *Vecturitrichia*). The description is the same as for the family *Vecturitrichaceae*. Type order: *Vecturitrichales* order nov**.**

**Description of *Modulibacteria* phylum nov.**

The phylum *Modulibacteria* is defined on a phylogenetic basis by comparative analysis of two KSB3 population genomes (UASB14 and 270, accession DF820455-DF820462 and DF820463-DF820483, respectively) and comparative 16S rRNA gene sequence from multiple terrestrial and aquatic habitats. Gram-negative bacteria possessing LPS in their cell envelopes. Some members have large (7-8Mb) genomes. Named classes include *Moduliflexia* and *Vecturitrichia.*
