# Supplementary material for: First genomic insights into members of a candidate bacterial phylum responsible for wastewater bulking
Source: PeerJ. 2015 Jan 27;3:e740. doi: 10.7717/peerj.740 (PMC4312070; doi:10.7717/peerj.740)
Supplement: Table S1 [file peerj-03-740-s016.docx]

**Supplementary Table S1 |** Shotgun sequencing statistics for UASB samples used for differential coverage banning, and relative representation of KSB3 genomes in thee data.

| Samples^a^ | Description | No. paired-end reads^b^ | No. bases reads for paired-end sequencing (Gb)^b^ | No. mate-pair reads^b^ | UASB14 abundance %^c^ | | UASB270 abundance %^c^ | |
| --- | --- | --- | --- | --- | --- | --- | --- | --- |
|  |  |  |  |  | based on amplicon sequencing | based on shotgun sequencing | based on amplicon sequencing | based on shotgun sequencing |
| A1 | Sludge taken at 25, December, 2012 | 61,998,374 | 14.7 | 29,866,508 | 4.64 | 9.98 | 0.26 | 0.1 |
| F1 | Flocculant part of sample A1 | 30,650,188 | 6.9 | - | - | 9.23 | - | 0.08 |
| G1 | Granular sludge part of sample A1 | 67,123,180 | 15.7 | - | - | 7.23 | - | 0.23 |
| A2 | Sludge taken at 16 September, 2010 | 80,896,216 | 18.9 | - | 3.45 | 10.39 | 0.22 | 0.98 |

a. All the sludge samples were obtained from a full-scale UASB reactor treating isomerized sugar processing wastewater. A1 and A2 samples represent whole sludge samples taken directly from the reactor system. F1 and G1 were gravimetrically separated from sample A1.

b. Sequencing was initially performed and described in Soo *et al.* 2014, and additional deep paired-end and mate-pair sequencing for the samples was performed in this study. Paired-end and mate-pair reads were generated using Illumina 2 x 250 cycles sequencing kits. The relative abundance of KSB3 populations based on 16S amplicon sequence were determined as an average of duplicate determination of 16S rRNA gene amplicon sequencing on the Illumina MiSeq platform (Caporaso et al. 2012).

c. The relative abundance based on shotgun sequencing was calculated using the 16S rRNA gene sequence close-reference OTU picking method in QIIME (http://qiime.org/tutorials/otu_picking.html; QIIME v1.6.0 (Caporaso et al*.* 2010); with the greengenes database (de-replicated dataset at 97%, March 2013, MacDonald et al. 2011) as reference with the following parameters: otu_picking_method, uclust_ref; similarity cutoff value, 0.95) with respective shotgun paired-end metagenome data.
