# Supplementary material for: First genomic insights into members of a candidate bacterial phylum responsible for wastewater bulking
Source: PeerJ. 2015 Jan 27;3:e740. doi: 10.7717/peerj.740 (PMC4312070; doi:10.7717/peerj.740)
Supplement: Table S2 — Summary of copy number anomalies in the 111 conserved single copy marker gene set (Dupont et al., 2012) found in the Modulibacteria (KSB3) genomes and most parsimonious explanations based on phylogenetic inference of gene neighborhoods. [file peerj-03-740-s017.docx]

**Supplementary Table S2 |** Summary of copy number anomalies in the 111 conserved single copy marker gene set (Dupont et al*.* 2012) found in the Modulibacteria (KSB3) genomes and most parsimonious explanations based on phylogenetic inference of gene neighborhoods.

|  |  |  | No. genes | | Most likely explanation^b^ |
| --- | --- | --- | --- | --- | --- |
| Gene family | Gene annotation^a^ | Size (aa) | UASB14 | UASB270 |  |
| TIGR00116 | translation elongation factor Ts | 293 | 0 | 0 | Truly absent in KSB3 lineage |
| TIGR00388 | glycine--tRNA ligase, alpha subunit | 293 | 0 | 0 | Truly absent in KSB3 lineage |
| TIGR00389 | glycine--tRNA ligase | 606 | 0 | 0 | Truly absent in KSB3 lineage |
| TIGR00409 | proline--tRNA ligase | 568 | 0 | 0 | Truly absent in KSB3 lineage |
| TIGR00775 | Na+/H+ antiporter, NhaD family | 420 | 0 | 0 | Truly absent in KSB3 lineage |
| TIGR02387 | DNA-directed RNA polymerase, gamma subunit | 619 | 0 | 0 | Truly absent in KSB3 lineage |
| TIGR00422 | valine--tRNA ligase | 863 | 0 | 1 | Gene missing from UASB14 draft genome |
| TIGR02432 | tRNA(Ile)-lysidine synthetase | 189 | 1 | 0 | Gene missing from UASB270 draft genome |
| TIGR02191 | ribonuclease III | 219 | 0 | 1 | Gene missing from UASB14 draft genome |
| PF01795 | MraW methylase family | 310 | 1 | 2 | Lateral transfer of additional homolog  into UASB270 lineage only |
| TIGR00418 | threonine--tRNA ligase | 565 | 1 | 2 | Lateral transfer of additional homolog  into UASB270 lineage only |
| TIGR02397 | DNA polymerase III, subunit gamma and tau | 355 | 2 | 2 | Gene duplication in the ancestor of UASB14 and UASB270 |
| PF00347 | Ribosomal protein L6 | 77 | 2 | 2 | Gene duplication in the ancestor of UASB14 and UASB20 |
| TIGR02027 | DNA-directed RNA polymerase, alpha subunit | 298 | 2 | 2 | Gene duplication in the ancestor of UASB14 and UASB20 |
| TIGR00362 | chromosomal replication initiator protein DnaA | 437 | 2 | 2 | Gene duplication in the ancestor of UASB14 and UASB20 |
| TIGR00468 | phenylalanine--tRNA ligase, alpha subunit | 324 | 2 | 2 | Lateral transfer of one homolog into the ancestor of  UASB14 and UASB270 |

a. For the analysis, all the scaffolds in each genome bin were first translated into six reading frames and hmmsearch in HMMER3 (Eddy 2011) was used to screen a set of 111 single copy marker genes conserved in most bacteria (Dupont *et al.* 2012) using CheckM (http://ecogenomics.github.io/CheckM) with the default settings. Putative missing genes in the gene set are further surveyed using BLASTP search against the predicted gene products from the KSB3 genomes. The gene products from putative multi-copy genes, which were detected using CheckM, and respective homologs found in finished bacterial and archaeal genomes in IMG (release 4.1, Markowitz et al. 2013) are aligned using hmmalign in HMMER3, and were subjected to phylogenetic analyses with FastTree with the default settings to see the phylogenetic positions of the multi-copy genes.

b. Duplicated genes in KSB3 lineage was verified based on their phylogenetic positions of each gene tree, where each copy of the homologues makes a clade with one of the relevant homologues in other genome bin, all the four homologues clustered together as a clade, indicating that these genes are duplicated in KSB3 lineage. To evaluate the identity of putative missing and contaminated genes with other known proteins from other organisms, the BLASTP search was performed in NCBI web site (http://blast.ncbi.nlm.nih.gov) against the non-redundant protein database (nr, all non-redundant GenBank CDS translations+PDB+SwissProt+PIR+PRF excluding environmental samples from WGS projects).
