# Supplementary material for: First genomic insights into members of a candidate bacterial phylum responsible for wastewater bulking
Source: PeerJ. 2015 Jan 27;3:e740. doi: 10.7717/peerj.740 (PMC4312070; doi:10.7717/peerj.740)
Supplement: Table S3 [file peerj-03-740-s018.docx]

**Supplementary Table S3 |** Taxon-outgroup configurations for phylogenetic inference based on concatenated marker gene sets.

| Outgroup configuration | Taxa used^a^ | No. ingroup | No. outgroup | No. marker genes | No. positions used | Inference methods | No. bootstraps |
| --- | --- | --- | --- | --- | --- | --- | --- |
| Config 1 | Bacteria + Archaea | 277 | 84 | 38 | 6,421 | RAxML (JTT+G), FastTree (JTT+CAT) | 100 |
| Config 2 | Bacteria | 277 | - | 83 | 17,723 | RAxML (JTT+G), FastTree (JTT+CAT) | 100 |
| Config 3 | Bacteria + Archaea including candidate bacterial phyla | 301 | 84 | 38 | 6,244 | RAxML (JTT+G), FastTree (JTT+CAT) | 100 |
| Config 4 | Bacteria including candidate bacterial phyla | 301 | - | 83 | 17,045 | RAxML (JTT+G), FastTree (JTT+CAT) | 100 |

a 275 bacterial and 84 archaeal isolate reference genomes representing their phyla were obtained from IMG (4.1, Markowitz et al. 2013). Twenty four draft genomes representing 17 additional bacterial candidate phyla were included in Configs 3 and 4.
