# Supplementary material for: First genomic insights into members of a candidate bacterial phylum responsible for wastewater bulking
Source: PeerJ. 2015 Jan 27;3:e740. doi: 10.7717/peerj.740 (PMC4312070; doi:10.7717/peerj.740)
Supplement: Table S4 [file peerj-03-740-s019.docx]

**Supplementary Table S4 |** Support for Modulibacteria (KSB3) monophyly and affiliation with potential sister phyla based on phylogenetic inferences with varying taxon configurations, inference methods and marker gene sets.

| Tree | Taxon-configuration | Treeing method | No. marker genes | Bootstrap value supporting monophyly of Modulibacteria % | Sister phyla with KSB3 clade | Bootstrap value supporting monophyly of KSB3 and sister phylum % |
| --- | --- | --- | --- | --- | --- | --- |
| 1 | Config 1 | RAxML (JTT+G) | 38 | 100 | *Nitrospinae* | 58 |
| 2 | Config 1 | FastTree (JTT+CAT) | 38 | 100 | *Acidobacteria* | 19 |
| 3 | Config 2 | RAxML (JTT+G) | 83 | 100 | *Nitrospinae* | 70 |
| 4 | Config 2 | FastTree (JTT+CAT) | 83 | 100 | *Acidobacteria* | 65 |
| 5 | Config 3 | RAxML (JTT+G) | 38 | 100 | *Nitrospinae* | 41 |
| 6 | Config 3 | FastTree (JTT+CAT) | 38 | 100 | NC10 | 28 |
| 7 | Config 4 | RAxML (JTT+G) | 83 | 100 | *Nitrospinae* | 35 |
| 8 | Config 4 | FastTree (JTT+CAT) | 83 | 100 | NC10 | 10 |
