# Supplementary material for: First genomic insights into members of a candidate bacterial phylum responsible for wastewater bulking
Source: PeerJ. 2015 Jan 27;3:e740. doi: 10.7717/peerj.740 (PMC4312070; doi:10.7717/peerj.740)
Supplement: Table S5 [file peerj-03-740-s020.docx]

**Supplementary Table S5 |** 16S rRNA-targeted fluorescence *in situ* hybridization (FISH) probes used in this study.

| Probe name^a^ | Target group | Probe Sequence (5' to 3') | Probe length (nt) | Target site (*E.coli* position) | Formamide (%) | Reference |
| --- | --- | --- | --- | --- | --- | --- |
| EUB338 | Domain Bacteria | GCTGCCTCCCGTAGGAGT | 18 | 338-355 | 10 | Amann *et al*. 1990 |
| EUB338-I | Domain Bacteria | GCAGCCTCCCGTAGGAGT | 18 | 338-355 | 10 | Daims *et al*. 1999 |
| EUB338-II | Domain Bacteria | GCAGCCACCCGTAGGTGT | 18 | 338-355 | 10 | Daims *et al*. 1999 |
| EUB338-III | Domain Bacteria | GCTGCCACCCGTAGGTGT | 18 | 338-355 | 10 | Daims *et al*. 1999 |
| ARC915 | Domain Archaea | GTGCTCCCCCGCCAATTCCT | 20 | 915-935 | 35 | Stahl *et al.* 1988 |
| 3KSB129 | Clone YM-1 | CCCATCCCGTGGTCGGTT | 18 | 129-136 | 30 | Yamada *et al*. 2007 |
| 3KSB1238 | Phylum KSB3 | CCGCTGTCCTCCCCATT | 17 | 1238-1255 | 25 | Yamada *et al*. 2007 |
| KSB3-703 | Phylum KSB3 | GACGTTCTTCCTGATCTC | 18 | 703-720 | 10 | Present study |
| KSB3-703 helper 1^b^ | Phylum KSB3 | CCGCTCCACCAGGAATTCCG | 20 | 672-693 | 10 | Present study |
| KSB3-703 helper 2^b^ | Phylum KSB3 | CCAGNNNGCCGCCTTCGCCAC | 21 | 722-741 | 10 | Present study |
| KSB3MAT644 | Class MAT-CR-H3-D11 | CGACACTCTAGCCCATCA | 18 | 644-661 | 10 | Present study |
| KSB3MAT644 helper 1^b^ | Class MAT-CR-H3-D11 | GTTTGCACCGCCCTTCCCC | 20 | 625-643 | 10 | Present study |
| KSB3MAT644 helper 2^b^ | Class MAT-CR-H3-D11 | ACCAGGAATTCCGNNTCCCCCT | 22 | 663-684 | 10 | Present study |

a. Probes were labeled with either Alex488 or Cy3 fluophores and probes with different fluophores are simultaneously used for dual probe FISH, as shown in **Fig. 1b**.

b. Helper probes were added equimolar with the fluorescent counterparts.

**References**

Amann, R. I. et al. 1990. Combination of 16S rRNA-targeted oligonucleotide probes with flow cytometry for analyzing mixed microbial populations. Appl Environ Microbiol 56:1919–1925.

Daims, H., Brühl, A., Amann, R., Schleifer, K. H. & Wagner, M. (1999). The domain-specific probe EUB338 is insufficient for the detection of all Bacteria: development and evaluation of a more comprehensive probe set. Syst Appl Microbiol 22:434–444.

Stahl, D. A., Flesher, B., Mansfield, H. R. & Montgomery, L. 1988. Use of phylogenetically based hybridization probes for studies of ruminal microbial ecology. Appl Environ Microbiol 54:1079–1084.

Yamada, T. et al. 2007. Characterization of filamentous bacteria, belonging to candidate phylum KSB3, that are associated with bulking in methanogenic granular sludges. ISME J 1:246–255.
