# Supplementary material for: First genomic insights into members of a candidate bacterial phylum responsible for wastewater bulking
Source: PeerJ. 2015 Jan 27;3:e740. doi: 10.7717/peerj.740 (PMC4312070; doi:10.7717/peerj.740)
Supplement: Table S6 [file peerj-03-740-s021.docx]

**Supplementary Table S6 |** tRNA genes found in the Modulibacteria (KSB3) genomes.

| Animo acid type | UASB14 | Distribution of amino acids in predicted gene products (%)^a^ |  | UASB270 | Distribution of amino acids in predicted gene products (%)^a^ |
| --- | --- | --- | --- | --- | --- |
| Ala | tRNA-Ala(ggc) x2, tRNA-Ala(tgc), tRNA-Ala(cgc) | 9.3 |  | tRNA-Ala(ggc), tRNA-Ala(tgc), tRNA-Ala(cgc) | 8.2 |
| Arg | tRNA-Arg(acg), tRNA-Arg(tcg), tRNA-Arg(ccg), tRNA-Arg(tct), tRNA-Arg(cct) | 5.8 |  | tRNA-Arg(acg), tRNA-Arg(tcg), tRNA-Arg(ccg), tRNA-Arg(tct), tRNA-Arg(cct) | 5.3 |
| Asn | tRNA-Asn(gtt) x4 | 3.6 |  | tRNA-Asn(gtt)^b^ | 3.6 |
| Asp | tRNA-Asp(gtc) | 5.2 |  | tRNA-Asp(gtc) | 5.0 |
| Cys | tRNA-Cys(gca) x2 | 1.1 |  | tRNA-Cys(gca) x2 | 1.1 |
| Gln | tRNA-Gln(ttg), tRNA-Gln(ctg) | 4.7 |  | not detected | 4.9 |
| Glu | tRNA-Glu(ttc), tRNA-Glu(ctc) | 4.7 |  | tRNA-Glu(ttc) x2, tRNA-Glu(ctc) | 4.9 |
| Gly | tRNA-Gly(gcc) x2, tRNA-Gly(tcc), tRNA-Gly(ccc) | 6.6 |  | tRNA-Gly(gcc) x2, tRNA-Gly(ccc) | 6.8 |
| His | tRNA-His(gtg) | 2.4 |  | tRNA-His(gtg) | 2.4 |
| Ile | tRNA-Ile(gat) x2 | 6.9 |  | tRNA-Ile(gat) | 7.3 |
| Leu | tRNA-Leu(taa), tRNA-Leu(caa), tRNA-Leu(gag), tRNA-Leu(tag), tRNA-Leu(cag) | 7.5 |  | tRNA-Leu(taa), tRNA-Leu(caa), tRNA-Leu(gag), tRNA-Leu(tag), tRNA-Leu(cag), | 7.4 |
| Lys | tRNA-Lys(ttt) | 4.8 |  | tRNA-Lys(ttt) x2 | 5.1 |
| Met | tRNA-Met(cat) x4 | 2.6 |  | tRNA-Met(cat) x2 | 2.5 |
| Phe | tRNA-Phe(gaa) | 4.6 |  | tRNA-Phe(gaa) | 4.6 |
| Pro | tRNA-Pro(ggg), tRNA-Pro(tgg), tRNA-Pro(cgg) x2 | 4.5 |  | tRNA-Pro(ggg), tRNA-Pro(tgg), tRNA-Pro(cgg) | 4.4 |
| Ser | tRNA-Ser(gga), tRNA-Ser(tga), tRNA-Ser(cga), tRNA-Ser(gct) | 8.7 |  | tRNA-Ser(gga), tRNA-Ser(tga) x2, tRNA-Ser(cga), tRNA-Ser(gct) | 8.9 |
| Sec | tRNA-seC(tca) | 0.3 |  | tRNA-seC(tca) | 0.3 |
| Thr | tRNA-Thr(ggt), tRNA-Thr(tgt), tRNA-Thr(cgt) | 5.5 |  | tRNA-Thr(tgt), tRNA-Thr(cgt) | 5.7 |
| Trp | tRNA-Trp(cca) | 1.2 |  | tRNA-Trp(cca) | 1.3 |
| Tyr | tRNA-Tyr(gta) | 3.3 |  | not detected | 3.5 |
| Val | tRNA-Val(gac), tRNA-Val(cac) | 6.6 |  | tRNA-Val(gac), tRNA-Val(cac) | 6.8 |

a. Distribution of amino acids (%) was estimated based on the counts of individual amino acids in all gene products from the genomes.

b. Not detected in original annotation, identified by BLAST analysis using UASB14 orthologs.
