# Supplementary material for: First genomic insights into members of a candidate bacterial phylum responsible for wastewater bulking
Source: PeerJ. 2015 Jan 27;3:e740. doi: 10.7717/peerj.740 (PMC4312070; doi:10.7717/peerj.740)
Supplement: Table S7 [file peerj-03-740-s022.docx]

**Supplementary Table S7 |** Inventory of putative glycoside hydrolases (GHs) identified in the Modulibacteria (KSB3) genomes.

| CAZy family^a^ | Known activity | Pfam/COG/cd domain | UASB14 | UASB270 |
| --- | --- | --- | --- | --- |
| **Cellulases** | | | | |
| GH5 | cellulase | pfam00150 | 2 | 3 |
| GH9 | endoglucanase | pfam00759 | 2 | 1 |
| GH10 | endo-xylanase | pfam00331 | 1 | 0 |
| GH28 | polygalacturonase | pfam00295 | 0 | 2 |
| GH74 | endoglucanase | smart00602 | 0 | 7 |
| **Endohemicellulases** | | | | |
| GH8 | endo-xylanase | pfam01270 | 0 | 1 |
| **Amylases** |  |  |  |  |
| GH13 | alpha-amylase | pfam00128 | 13 | 7 |
| GH57 | alpha-amylase | pfam03065 | 6 | 1 |
| **Debranching enzymes** | | | | |
| GH51 | alpha-L-arabinofuranosidase | COG3534 | 2 | 2 |
| GH78 | alpha-L-rhamnosidase | pfam05592 | 1 | 0 |
| **Amino sugar-degrading enzymes** | | | | |
| GH20 | beta-hexosaminidase | pfam00728 | 1 | 1 |
| GH23 | lysozyme | cd00254 | 11 | 9 |
| GH24 | lysozyme | cd00737 | 0 | 1 |
| GH25 | lysozyme | pfam01183 | 0 | 2 |
| GH46 | chitosanase | cd00978 | 1 | 0 |
| GH73 | peptidoglycan hydrolase | pfam01832 | 2 | 0 |
| GH103 | peptidoglycan lytic transglycosylase | TIGR02283 | 2 | 2 |
| GH109 | alpha-N-acetylgalactosaminidase | pfam01408 | 18 | 29 |
| GH114 | endo-alpha-1,4-polygalactosaminidase | pfam03537 | 0 | 1 |
| **Oligosaccharide-degrading enzymes** | | | | |
| GH1 | beta-glucosidase | pfam00232 | 3 | 1 |
| GH1 | beta-galactosidase | COG3250 | 6 | 4 |
| GH3 | beta-glucosidase | pfam00933 | 8 | 2 |
| GH4 | alpha-glucosidase | pfam02056 | 4 | 6 |
| GH29 | alpha-L-fucosidase | pfam01120 | 2 | 0 |
| GH31 | alpha-glucosidase | pfam01055 | 4 | 2 |
| GH32 | invertase | pfam00251 | 1 | 0 |
| GH33 | sialidase | cd00260 | 1 | 1 |
| GH35 | beta-galactosidase | pfam01301 | 1 | 0 |
| GH36 | alpha-galactosidase | COG3345 | 1 | 0 |
| GH37 | alpha,alpha-trehalase | pfam01204 | 1 | 0 |
| GH38 | alpha-mannosidase | pfam01074 | 1 | 2 |
| GH42 | beta-galactosidase | pfam02449 | 1 | 0 |
| GH43 | beta-xylosidase | pfam04616 | 6 | 0 |
| GH63 | alpha-glucosidase | PRK10137 | 1 | 0 |
| GH65 | alpha,alpha-trehalase | pfam03632 | 0 | 1 |
| GH76 | alpha-1,6-mannanase | pfam03663 | 1 | 1 |
| GH94 | cellobiose phosphorylase | COG3459 | 1 | 1 |
| GH100 | invertase | pfam04853 | 0 | 1 |
| GH120 | beta-xylosidase | pfam07602 | 0 | 1 |
| Total GHs |  |  | 105 | 92 |
| %GHs in total ORFs |  |  | 1.75 | 1.31 |

a. GHs are grouped into functional categories (Alcaide et al*.* 2012). Both partial and full-length sequences are included.
