# Supplementary material for: First genomic insights into members of a candidate bacterial phylum responsible for wastewater bulking
Source: PeerJ. 2015 Jan 27;3:e740. doi: 10.7717/peerj.740 (PMC4312070; doi:10.7717/peerj.740)
Supplement: Table S8 [file peerj-03-740-s023.docx]

**Supplementary Table S8 |** Inventory of Modulibacteria (KSB3) genes putatively involved in environmental signaling.

| Protein family | | COG domain | Pfam domain | No. in UASB14^a^ | |  | No. in UASB270^a^ | |
| --- | --- | --- | --- | --- | --- | --- | --- | --- |
|  |  |  |  | COG | Pfam |  | COG | Pfam |
| Transmembrane sensor molecules^b^ | |  |  |  |  |  |  |  |
|  | signal transduction histidine kinase | COG0642 | PF00512^d^ | 121 (36) | 141 |  | 92 (33) | 115 |
|  | adenylate/diguanylate cyclase | COG2114 | PF00211^d^ | 17 (8) | 17 |  | 12 (4) | 18 |
|  | methyl-accepting chemotaxis protein | ` | PF00015^d^ | 87 (87) | 80 |  | 69 (69) | 71 |
|  | GGDEF | COG2199 | PF00990^d^ | 8 (2) | 14 |  | 9 (3) | 20 |
|  | HD-GYP | COG2206 | - | 2 (0) | - |  | 3 (0) | - |
|  | serine/threonine protein kinase | COG0515 | PF00069^d^ | 11 (1) | 18 |  | 9 (2) | 19 |
|  | CHASE2 | COG4252 | - | 0 | - |  | 1 | - |
|  | CHASE4 | COG3322 | - | 1 | - |  | 2 | - |
|  | All sensors |  |  | 247 (135) | 270 |  | 197 (114) | 243 |
| All DNA-binding response regulators | |  |  |  |  |  |  |  |
|  | AraC | COG4753 | - | 3 | - |  | 2 | - |
|  | NarL | COG2197 | PF00196 | 7 | 7 |  | 4 | 7 |
|  | OmpR | COG0745 | - | 24 | - |  | 19 | - |
|  | NtrC | COG2204 | - | 52 | - |  | 49 | - |
|  | LytTR | COG3279 | - | 0 | - |  | 0 | - |
|  | Fis | COG4567 | PF02954 | 0 | 37 |  | 0 | 37 |
|  | All CheY-X response regulators^c^ |  | PF00072^d^ | 131 | 250 |  | 116 | 219 |
| Chemotaxis related genes | |  |  |  |  |  |  |  |
|  | CheA | COG0643 | - | 17 | - |  | 19 | - |
|  | CheB | COG2201 | - | 8 | - |  | 16 | - |
|  | CheD | COG1871 | - | 1 | - |  | 1 | - |
|  | CheR | COG1352 | - | 7 | - |  | 8 | - |
|  | CheY | COG0784 | - | 20 | - |  | 19 | - |
|  | CheW | COG0835 | - | 11 | - |  | 10 | - |

a. Counts are from either RPS-BLAST searches against COG PSSMs from the CDD database (Marchler-Bauer et al. 2013) using an e-value cutoff of 0.01 with the top hit retained for each protein domain (values in COG column), or motif analysis with PFAM (Punta et al. 2012) using HMMR3 (Eddy 2011) and PfamScan using e-value cutoff of 0.01 (values in Pfam column).

b. Total number of proteins containing each signaling domain is given, and the subset predicted to be transmembrane sensor proteins containing at least one transmembrane segment (estimated by using TMHMM Server v. 2.0, Moller et al. 2002) is shown in parentheses for COG counts.

c The number of all CheY-X response regulators is the sum of all two-domain response regulators, including proteins containing either COG3437, COG3706, and COG3947 domains for COG counts, or all proteins containing the PF00072 domain for Pfam counts.

d These six Pfam domains form the basis of the environmental signaling analyses shown in **Fig 5** and **Supplementary Fig. S13** according to Galperin 2004 and 2010.
