# Supplementary material for: First genomic insights into members of a candidate bacterial phylum responsible for wastewater bulking
Source: PeerJ. 2015 Jan 27;3:e740. doi: 10.7717/peerj.740 (PMC4312070; doi:10.7717/peerj.740)
Supplement: Table S9 [file peerj-03-740-s024.docx]

**Supplementary Table S9 |** Bacteria ranked by proportion of encoded signaling proteins.

|  | Organism | Phylum/class^a^ | IMG genome ID | No. of genes^b^ | % of total ORFs | No. of total ORFs |
| --- | --- | --- | --- | --- | --- | --- |
| **Histidine kinases (COG0642)** | | | | | | |
|  | *Stigmatella aurantiaca* DW4/3-1 | Deltaproteobacteria | 649633096 | 142 | 1.7 | 8590 |
|  | *Oscillatoria nigro-viridis* PCC 7112 | Cyanobacteria | 2503982035 | 140 | 2 | 6925 |
|  | *Corallococcus coralloides* DSM 2259 | Cyanobacteria | 2512564078 | 133 | 1.7 | 8033 |
|  | *Microcoleus* sp. PCC 7113 | Cyanobacteria | 2509276031 | 128 | 1.9 | 6734 |
|  | UASB14 | Modulibacteria (KSB3) | - | 121 | 2 | 5989 |
|  | *Desulfovibrio magneticus* RS-1 | Deltaproteobacteria | 644736352 | 117 | 2.5 | 4700 |
|  | *Myxococcus fulvus* HW-1 | Deltaproteobacteria | 650716065 | 116 | 1.5 | 7510 |
|  | *Myxococcus xanthus* DK 1622 | Deltaproteobacteria | 637000186 | 106 | 1.4 | 7369 |
|  | UASB270 | Modulibacteria (KSB3) | - | 92 | 1.3 | 7048 |
|  | *Magnetococcus* sp. MC-1 | Alphaproteobacteria | 639633036 | 87 | 2.3 | 3758 |
| **Methyl-accepting proteins (COG0840)** | | | | | | |
|  | *Azospirillum* sp. B510 | Alphaproteobacteria | 646311907 | 88 | 1.4 | 6309 |
|  | UASB14 | Modulibacteria (KSB3) | - | 87 | 1.5 | 5989 |
|  | *Clostridium saccharoperbutylacetonicum* ATCC 27021 | Firmicutes | 2519899526 | 73 | 1.2 | 5843 |
|  | *Herbaspirillum seropedicae* SmR1 | Betaproteobacteria | 648028033 | 70 | 1.4 | 4874 |
|  | UASB270 | Modulibacteria (KSB3) | - | 69 | 1 | 7048 |
|  | *Rhodospirillum photometricum* DSM 122 | Alphaproteobacteria | 2513237204 | 67 | 2 | 3281 |
|  | *Clostridium beijerinckii* NCIMB 8052 | Firmicutes | 640753016 | 65 | 1.3 | 5100 |
|  | *Magnetospirillum magneticum* AMB-1 | Alphaproteobacteria | 637000155 | 63 | 1.4 | 4559 |
|  | *Azospirillum lipoferum* 4B | Alphaproteobacteria | 2511231221 | 62 | 1 | 6094 |
|  | *Desulfovibrio magneticus* RS-1 | Deltaproteobacteria | 644736352 | 61 | 1.3 | 4700 |

a. Phylum level assignment is shown, except for that class level taxonomic names are shown for Proteobacteria.

b. The numbers are from the results of RPS-BLAST searches against COG PSSMs from the CDD database (Marchler-Bauer et al. 2013) using an e-value cutoff of 0.01 with the top hit retained for each protein domain for each genome obtained from IMG (release 4.1, Markowitz et al*.* 2013).
